# Predictive Value of Preoperative Left Atrial Strain Parameters on Postoperative Atrial Fibrillation in Adults Undergoing Cardiac Surgery: A Systematic Review and Meta-Analysis

**DOI:** 10.1093/icvts/ivag035

**Published:** 2026-02-13

**Authors:** Satyajeet Misra, Devishree Das, Tarun Kumar Patra, Deepak Prakash Borde, Anand Srinivasan

**Affiliations:** Department of Anesthesiology and Critical Care, All India Institute of Medical Sciences (AIIMS), Bhubaneswar 751019, Odisha, India; Department of Anesthesiology (Burn Centre), AIIMS, Bhubaneswar 751019, Odisha, India; Department of Anesthesiology (Burn Centre), AIIMS, Bhubaneswar 751019, Odisha, India; Department of Cardiac Anesthesia, Ozone Anesthesia Group, Aurangabad 431001, Maharashtra, India; Department of Pharmacology, AIIMS, Bhubaneswar 751019, Odisha, India

**Keywords:** cardiac surgery, left atrial strain, postoperative atrial fibrillation

## Abstract

**Objectives:**

Preoperative left atrial (LA) strain parameters measured by 2-dimensional speckle tracking echocardiography have been used to predict postoperative atrial fibrillation (POAF) after cardiac surgery. The aim of this meta-analysis was to determine whether preoperative LA strain parameters predict POAF after cardiac surgery.

**Methods:**

PubMed, Embase, Cochrane database, and Google Scholar were searched manually until January 31, 2025. Studies where preoperative LA strain was used to predict POAF following cardiac surgery in adults were considered. Reviews, case series, case reports, and studies where patients were in preoperative atrial fibrillation were excluded.

**Results:**

Twenty-four observational studies involving 2242 patients were included. Preoperative LA reservoir strain was significantly lower in patients with POAF vs those without POAF (standardized mean difference [SMD] −2.37; 95% confidence interval [CI] −3.87 to −0.88; *I*^2^ = 94.5%). Preoperative LA conduit (SMD −0.73; 95% CI, −1.06 to −0.39; *I*^2^ = 41.5%) and contraction (SMD −1.04; 95% CI, −1.81 to −0.27; *I*^2^ = 92.2%) strain were significantly lower in patients with POAF while preoperative LA reservoir, conduit, and contraction strain rates were not different in patients with POAF vs no POAF. Meta regression for heterogeneity in reservoir strain was significant for gender, vendor platform, and filling pressures (E/e’). The cut-off value of LA reservoir strain for predicting POAF was 22 to 25% (area under curve 0.69, specificity 0.679 [95% CI, 0.645 to 0.711], sensitivity 0.713 [95% CI, 0.675 to 0.743]).

**Conclusions:**

Preoperative LA reservoir, conduit, and contraction strain predict POAF in adults undergoing cardiac surgery.

**Prospero registration no:**

CRD42024606011.

## Graphical Abstract

**Figure:**
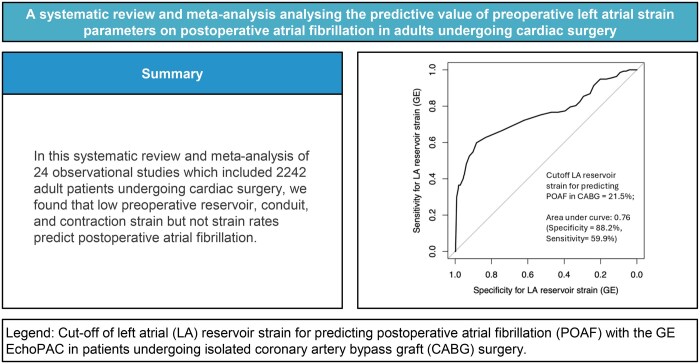

## INTRODUCTION

Postoperative atrial fibrillation (POAF) is one of the most common arrhythmias following cardiac surgery, with an incidence of 20% to 50%, depending on the definitions used or the type of surgical operations performed.^1–3^ It may result in haemodynamic instability, low cardiac output, thromboembolic events, stroke, and morbidity as well as mortality. POAF typically occurs on second to fourth postoperative day, often having transient episodes with a self-limiting course.^4^

Age, previous history of atrial fibrillation (AF), cardiac failure, renal failure, and chronic obstructive pulmonary disease are risk factors for POAF.^5^ Re-entry phenomena and ectopic firing due to triggered activity are the major mechanisms for development of POAF.^6^^,^^7^ Surgical stress, activation of the sympathetic systems as well as renin-angiotensin-aldosterone system, and extracorporeal induced systemic inflammation constitute the main perioperative triggers for POAF.^8^ Accuracy of preoperative risk stratification models for POAF is limited.^9^^,^^10^ This essentially implies that all patients may end up getting preventive treatment, and not necessarily those at increased risk of POAF.

Recently, various newer echocardiographic modalities like left atrial (LA) volume index (LAVI) and LA reservoir strain have been described to predict POAF.^11^^,^^12^ LA reservoir strain by 2-dimensional speckle tracking echocardiography (2D STE) is an angle and load independent parameter which indicates LA compliance. LA reservoir strain may decrease earlier compared to increase in LAVI in patients with reduced LA cardiopathy.^12^

A previous meta-analysis outlined various transthoracic indices for predicting POAF, but essentially focused on LAVI.^13^ It included only 5 studies reporting LA reservoir strain without analysing other strain parameters. In addition, cut-off values of LA strain were not provided for prediction of POAF. Following this review,^13^ there have been several recent studies exploring the predictive value of LA strain parameters in POAF. Therefore, this updated systematic review and meta-analysis was carried out to synthesize the current evidence of LA strain parameters for prediction of POAF following adult cardiac surgery.

## METHODS

### Search strategy

We followed the Meta-analysis Of Observational Studies in Epidemiology (MOOSE) 2021 guidelines for this review.^14^ The protocol was registered in the prospective register of systematic reviews (PROSPERO) (CRD42024606011). The research question was framed according to the PEO format, where P was “Population” (adults undergoing cardiac surgery), E was “Exposure” (preoperative LA strain parameters), and O was “Outcome” (POAF).^15^

### Search engines

A systematic manual literature search was conducted in PubMed, Embase, Cochrane database, and Google Scholar until January 31, 2025 (**Table S1**). Additionally, citation tracking was performed to identify more publications. Search terms included a combination of Medical Subject Headings (MeSH) terms and free-text keywords such as “postoperative atrial fibrillation,” “cardiac surgery,” “LA strain,” “strain rate,” “reservoir strain,” “contraction strain,” and “conduit strain.” Searches were combined using AND/OR options with filters applied for English language, human subjects (age ≥ 18 years), and clinical trials or observational studies.

### Study selection

Titles and abstracts of retrieved citations were initially independently screened by 4 authors (D.D., T.K.P., D.P.B., A.S.) to assess suitability for potential inclusion. This was followed by full-text evaluation, which was independently conducted by 3 authors (D.D., T.K.P., and D.P.B.). Disagreements regarding eligibility of an article/study were discussed and resolved by mutual agreement and discussion with the senior most author (SM). Data were extracted and entered in Microsoft excel (Microsoft Excel for Mac, version 16.95.1). For missing or ambiguous data, authors were contacted via email.

### Inclusion and exclusion criteria

Studies involving adult patients undergoing cardiac surgery reporting the incidence of POAF as primary or secondary outcome and where preoperative LA strain parameters were recorded by 2D STE were included. Conference abstracts, editorials, commentaries, review articles, case reports, case series, and publications in language other than English were excluded. We further excluded studies which enrolled patients with preoperative AF and those where tissue Doppler imaging was used for LA strain analysis.

### Definitions

- POAF: Any episode of AF lasting for any duration within 30 days of index cardiac surgery and whether treated or not was considered as POAF.
- LA reservoir strain: Systolic strain, peak positive strain, or peak longitudinal strain were considered as LA reservoir strain.
- LA conduit strain: Early diastolic strain was considered as LA conduit strain.
- LA contraction strain: Booster strain, late diastolic strain, late atrial strain, peak negative strain, and pump strain were considered as LA contraction strain.
- Strain rate: Strain parameters indexed to time were considered as respective strain rates.

### Study objectives

The primary objective was to determine whether preoperative LA reservoir strain predicts POAF in adults undergoing cardiac surgery.

The secondary objectives were:

- To determine whether LA conduit and contraction strain predict POAF.
- To determine whether LA strain rates predict POAF.
- To determine the cut-off value of preoperative LA functional and structural parameters in predicting POAF.

### Risk of bias assessment

The Newcastle Ottawa scale was used to assess the risk of bias of the included studies by 2 authors (D.D. and T.K.P.). Conflicts were resolved by a third author (A.S.). The scale is developed for assessing the quality of case-control and cohort studies.^16^ Assessment is made across 8 domains, including representativeness of the exposed cohort, selection of the non-exposed cohort, ascertainment of exposure, demonstration that outcome of interest was not present at start of study, comparability of the cohorts on the basis of design or analysis controlled for confounders, assessment of outcome, whether the outcome follow-up was long enough, and adequacy of follow-up.^16^ The maximum score awarded is 9, with lower scores representing higher bias.

### Certainty of evidence

The certainty of evidence was assessed by the Grading of Recommendations, Assessment, Development, and Evaluation (GRADE) method for the primary objective, that is, LA reservoir strain by 2 authors (T.K.P. and A.S.). Conflicts were resolved by a third author (D.P.B.). The GRADE approach assesses the certainty of evidence across 5 domains: methodological flaws of the studies (eg, risk of bias), heterogeneity of results across studies (eg, inconsistency), generalizability of findings (eg, indirectness), precision of the estimates (narrow confidence intervals [CIs]), and publication bias. Certainty of evidence ranges from high to very low.^17^

### Statistical analysis

The meta-analysis was performed in R (version 4.4) using the “meta” package. The various LA strain parameters, which are continuous variables, were analysed by calculating pooled standardized mean differences (SMDs) and their 95% CI using the random effects model (Der Simonian-Laird). SMDs of LA reservoir strain were also analysed according to the incidence of POAF reported in various studies. The prediction intervals were also calculated for the pooled parameters in the meta-analysis. Heterogeneity was assessed with the Cochrane Q-test and *I*^2^ statistics with a significant cut-off value of *P* < .05 and *I*^2^ > 50%, respectively. To explore the patterns of heterogeneity in reservoir strain, a leave-one-out study analysis was performed. Subgroup and multivariate meta-regression analyses were performed for between-study differences that could contribute to overall heterogeneity. Subgroups were defined based on the possible contributors to between-study differences (types of cardiac surgery, and vendor specific software used for strain analysis). For meta-regression, the variables considered were left ventricle (LV) global longitudinal strain (LV GLS), LAVI, LV filling pressure (average E/e’), gender, left ventricular ejection fraction (LVEF), age, vendor, the definition of POAF, sample size, and type of surgery used in various studies. Publication bias was assessed with funnel plot and Egger’s test. For the other LA strain parameters and LAVI, a leave-one-out study analysis was performed to explore the effect on heterogeneity.

Receiver operator characteristic (ROC) analyses were performed to identify a threshold value in the various LA parameters (strain and LAVI) that can differentiate between the presence or absence of POAF. For the ROC analyses, apart from the threshold values, the area under curve (AUC), sensitivity, and specificity were calculated for all the parameters evaluated. The ROC analysis was performed based on the descriptive statistical values reported in the included studies. When individual studies explicitly reported their optimal cut-off values in their publications, these were used directly. When studies did not report specific cut-off values, thresholds were calculated as the midpoint between group means. The ROC analysis was performed using the “pROC” package in R. The 2-tailed *P*-value cut-off for statistical significance was set at < .05 for all analyses. Diagnostic accuracy was assessed using bivariate random-effects meta-analysis, which jointly estimated pooled sensitivity and specificity while accounting for within-study correlation. Individual study 2 × 2 contingency tables were derived from summary statistics, and diagnostic odds ratios were calculated from pooled estimates.

## RESULTS

### Study characteristics

**Figure 1.**
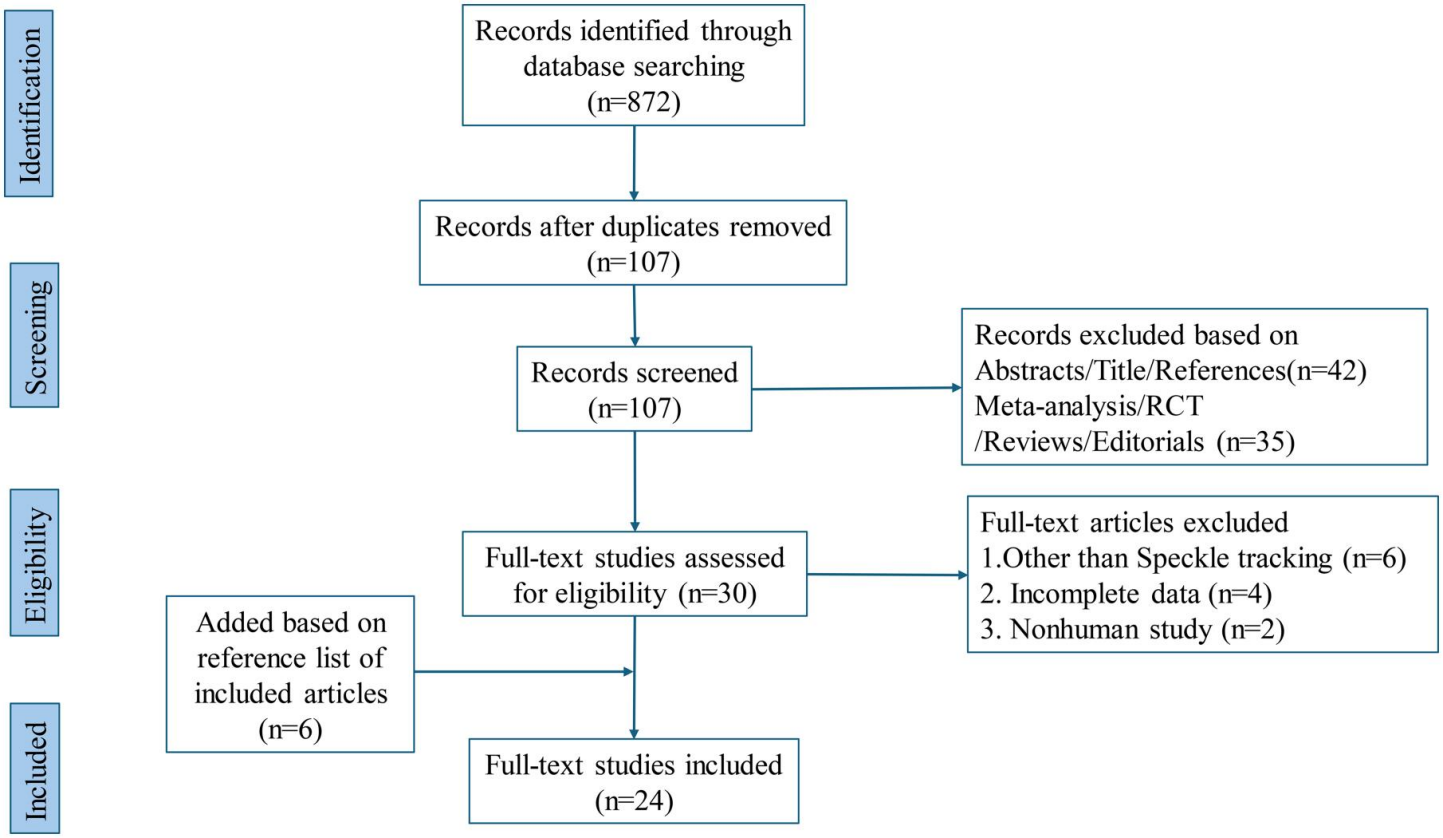
Meta-Analysis of Observational Studies in Epidemiology (MOOSE) Flowchart. Abbreviation: RCT, randomized controlled trial.

We identified 24 studies involving 2242 patients that were eligible for the systematic review and meta-analyses (**Figure 1**).^18–41^ Summary of the 24 included studies is presented in **Table 1**. Twenty-two studies were prospective studies,^18–35^^,^^37–39^^,^^41^ while 2 studies were retrospective.^36^^,^^40^ Sixteen studies were in patients undergoing coronary artery bypass graft (CABG),^18–20^^,^^23^^,^^25–27^^,^^29^^,^^32–36^^,^^39–41^ 5 studies were carried out in patients undergoing aortic valve (AV) surgery,^22^^,^^24^^,^^28^^,^^30^^,^^38^ 2 studies were in mitral valve (MV) surgery,^21^^,^^31^ while 1 study included patients undergoing combined CABG and valve surgery.^37^

**Table 1.** Characteristicsof the Included Studies

Sl No	Authors	Study design	Surgery	No. of patients	Mean age (years)	Sex (male)	LV EF (%)
1	Tayyareci et al.^18^	Prospective	On-pump CABG	96	POAF = 68.6 + 8.2, no POAF = 61.3 + 10.4	POAF = 88%, no POAF = 79%	POAF = 53.4 + 7.1, no POAF = 53.6 ± 6.9
2	Gabrielli et al.^19^	Prospective	On-pump CABG	70	POAF = 70 + 2, no POAF = 62 ± 2	POAF = 66%, no POAF = 73%	POAF = 61 + 4, no POAF = 65 ± 6
3	Her et al.^20^	Prospective	On-pump CABG	53	POAF = 71 + 5, no POAF = 64 ± 10	–	POAF = 63.5 + 10.8, no POAF = 56.4 ± 14.5
4	Candan et al.^21^	Prospective	MV surgery (30 MV repair, 23 MVR)	53	POAF = 54.6 + 12.2, no POAF = 42.2 ± 12.5	POAF = 40%, no POAF = 47.4%	POAF = 66.1 + 9, no POAF = 64 ± 6.3
5	Imanishi et al.^22^	Prospective	AVR	27	POAF = 78 + 10, no POAF = 75 ± 6	POAF = 20%, no POAF = 42%	POAF = 61 + 13, no POAF = 66 ± 8
6	Parsaee et al.^23^	Prospective (cross sectional study)	On-pump CABG	150	POAF = 67.11 ± 9.05, no POAF = 59.19 ± 8.59	POAF = 73.7%, no POAF = 75.6%	POAF = 41.63 ± 10.87, no POAF = 42.78 ± 9.59
7	Cameli et al.^24^	Prospective	AVR	76	POAF = 71.5 ± 10.1, no POAF = 66.5 ± 12.1	POAF = 65.9%, no POAF = 68.7%	POAF = 58.2 ± 7.9, no POAF = 58.6 ± 7.9
8	Verdejo et al.^25^	Prospective (case-control)	On-pump CABG	70	–	–	–
9	Ozben et al.^26^	Prospective	On-pump CABG	48	POAF = 64.5 + 7.7, no POAF = 60.5 ± 9.2	POAF = 61.5%, no POAF = 88.6%	POAF = 54.5 + 5.8, no POAF = 56.6 ± 5.9
10	Başaran et al.^27^	Prospective	CABG	90	POAF = 64.3 ± 7.4, no POAF = 58.7 ± 10.1	POAF = 69%, no POAF = 82%	POAF = 62.63 ± 11.93, no POAF = 61.77 ± 16.74
11	Pernigo et al.^28^	Prospective	AVR	60	POAF = 73.6 + 7.6, no POAF = 69.3 ± 8.1	POAF = 50%, no POAF = 50%	POAF = 58 + 10, no POAF = 60 ± 9
12	Aksu et al.^29^	Prospective	CABG	60	POAF = 68.14 ± 12.16, no POAF = 67.66 ± 11.34	POAF = 75%, no POAF = 72%	POAF = 49 ± 10, no POAF = 51.93 ± 9.69
13	Pessoa-Amorim et al.^30^	Prospective	AVR	114	–	–	–
14	Lisi et al.^31^	Prospective	MV repair	37	–	–	POAF = 59.7 ± 6.7, no POAF = 60.8 ± 3.9
15	Sabry et al.^32^	Prospective	CABG	100	POAF = 65.3 ± 5.3, no POAF = 56.9 ± 7.8	POAF = 72.72%, no POAF = 66.6%	POAF = 56 ± 7, no POAF = 60 ± 6
16	Rizvi et al.^33^	Prospective	CABG	90	POAF = 72.4 ± 10.8, no POAF = 67.3 ± 10.7	POAF = 77.4%, no POAF = 66.7%	POAF = 58.3 ± 9.4%, no POAF = 55.4 ± 11.5
17	Darweesh et al.^34^	Prospective	On-pump CABG	84	POAF = 61 ± 5.89, no POAF = 56.58 ± 6.97	–	POAF = 51.25 ± 10.77, no POAF = 58.35 ± 7.88
18	Abdelrazek et al.^35^	Prospective	On-pump CABG	89	POAF = 58.7 ± 9.2, no POAF = 55 ± 6.4	POAF = 64.3%, no POAF = 65.6%	POAF = 61.5 ± 5.3, no POAF = 61.8 ± 7.9
19	Kislitsina et al.^36^	Retrospective	On-pump CABG	211	POAF = 66.3 + 9.3, no POAF = 66.1 ± 9.3	POAF = 89%, no POAF = 89%	POAF = 59.4 ± 9.4, no POAF = 55.3 ± 11.3
20	Dalos et al.^37^	Prospective	CABG + Valve	124	POAF = 73.03 + 7.69, no POAF = 68.53 + 11.66	POAF = 57.4%, no POAF = 67.7%	POAF = 58.33 + 3.81, no POAF = 56.67 + 7.57
21	Wedin et al.^38^	Prospective	AVR	227	–	–	–
22	Pastore et al.^39^	Prospective	On-pump CABG	310	POAF = 70.6 + 7.8, no POAF = 65.9 ± 8.6	POAF = 84%, no POAF = 80%	POAF = 47.7 + 12.0.3, no POAF = 51 ± 10.6
23	Borde et al.^40^	Retrospective	Off-pump CABG	103	POAF = 66 + 6, no POAF = 61 ± 10	POAF = 95.8%, no POAF = 77.2%	POAF = 44 + 8, no POAF = 46 ± 9
24	Granchietti et al.^41^	Prospective	On pump CABG	100	POAF = 69.6 ± 9.9, no POAF = 67.1 ± 7.9	POAF = 92.6 %, no POAF = 87.7%	POAF = 48.2 ± 10.2, no POAF = 51.7 ± 10.4

Abbreviations: AVR, aortic valve replacement; CABG, coronary artery bypass grafting; LVEF, left ventricular ejection fraction; MVR, mitral valve replacement; POAF, postoperative atrial fibrillation.

Eleven studies defined POAF as new-onset AF lasting >30 seconds,^18^^,^^20^^,^^22^^,^^24^^,^^28^^,^^32–35^^,^^38^^,^^39^ 6 studies considered a duration >15 minutes,^19^^,^^21^^,^^25–27^^,^^36^ and 1 study defined AF lasting > 5 minutes^23^ as POAF. Six studies^29–31^^,^^37^^,^^40^^,^^41^ did not give a time duration for defining POAF. Seven hundred and forty-one patients (33.05%) developed POAF, which is similar to the reported incidence of POAF after cardiac surgery in literature (20%-50%).^1^^,^^42^ Of the patients developing POAF, 69% were male. The mean (SD) for age and LVEF of patients developing POAF were 67.7 (8.2) y and 55.8 (8.7) % respectively. **Table 2** outlines the characteristics of LA strain parameters and LAVI in predicting POAF.

**Table 2.** Reported Echocardiographic Parameters in Predicting POAF

Sl No	Authors	Incidence of POAF (%)	POAF definition	Follow up period	LA reservoir strain (%) or strain rate (per second) value in POAF vs no POAF	Cut-off for LA Reservoir strain to predict POAF (%)	LA contraction strain (%)/strain rate (per second)	LA conduit strain (%)/strain rate (per second)	**LA volume index (mL/m** ^2^ **)**	Strain analysis software	ECHO machine
1	Tayyareci et al (2010)^18^	26.04	>30 seconds	Postoperative Until discharge	POAF = 39.9 ± 14.0%, no POAF = 50.1 ± 15.9% POAF = 1.5 ± 0.8 per second, no POAF = 1.8 ± 0.6 per second	44	– (TDI values)	POAF = 1.9 ± 0.7per second, no POAF = 2.1 ± 0.8per second	POAF = 35.10 ± 6.7, no POAF = 25.8 ± 5.9	Syngo VVI, Siemens Medical Solutions	Siemens, Sequoia, C256
2	Gabrielli et al (2011)^19^	25.71	>15 minutes	Postoperative Until 7 days	POAF = 10 + 1%; no POAF = 24 + 1% POAF = 0.6 ± 0.1 per second, no POAF = 1.2 ± 0.1 per second	–	POAF = −0.6 ± 0.1 per second, no POAF= −1.8 ± 0.12 per second	–	POAF = 30 ± 4, no POAF = 23 ± 1	GE EchoPAC	VIVID7, GEMedical Health, Horten, Norway
3	Her et al (2013)^20^	24.52	>30 seconds	Postoperative Until discharge	POAF = 25.4 + 10.4%; no POAF = 36.8 + 7.6% POAF = 1.2 ± 0.6 per second, no POAF = 1.6 ± 0.8 per second	27.70	–	POAF = −1.4 ± 0.8 per second, no POAF = −1.8 ± 0.8 per second	POAF = 32.6 ± 5.1, no POAF = 27.3 ± 7.2	GE EchoPAC	GE Vivid 7
4	Candan et al (2013)^21^	28.30	>15 minutes	Postoperative Until 7 days	POAF = 13.9 + 3.8%; no POAF = 24.8 + 7.3%	–	POAF = 7.6 ± 1.95%, no POAF = 11.3 ± 3.5 %	–	POAF = 72.6 ± 9.2, no POAF= 52.6 ± 14.2	GE EchoPAC	GE Vivid7
5	Imanishi et al (2014)^22^	55.56	>30 seconds	Postoperative until 14.5 + 5.7 days	POAF = 0.80 + 0.41per second; no POAF = 1.3 + 0.47 per second	LAS rate 0.79 per second	POAF = 0.78 ± 0.26 per second, no POAF = 1.1 ± 0.22 per second	POAF = 0.33 ± 0.2 per second, no POAF = 0.46 ± 0.21 per second	POAF = 66 ± 20, no POAF = 58 ± 17	Toshiba Ultra Extend	Aplio Artida; Toshiba Medical Systems
6	Parsaee et al (2014)^23^	12.67	>5 minutes	Postoperative Until 5 days	POAF= −1.55 ± 0.87 per second, no POAF= −1.51 ± 0.92 per second	–	POAF = 1.36 ± 0.70 per second, no POAF = 1.32 ± 0.62 per second	POAF= −0.76 ± 0.58 per second, no POAF= −1.61 ± 0.62 per second	POAF = 30.48 ± 10.20, no POAF = 40.29 ± 13.445	Xstraine licence software	Mylab60, Esaote
7	Cameli et al (2014)^24^	19.74	>30 seconds	Postoperative Until discharge	POAF = 22.5 + 7.1; no POAF = 33.6 + 9.5	16.90	–	–	POAF = 47.9 ± 17.1, no POAF = 41.6 ± 15.2	GE EchoPAC	Vivid 7, GE
8	Verdejo et al (2016)^25^	38.57	>15 minutes	Postoperative Until 3 days	POAF= 10.0 ± 1.1%, no POAF = 24.0 ± 1.2% POAF = 0.6 ± 0.01 per second, no POAF = 1.2 ± 0.01 per second	–	POAF= −0.6 ± 0.1 per second, no POAF = 1.8 ± 0.12 per second	–	–	GE EchoPAC	VIVID 7; GE Medical Systems
9	Ozben et al (2016)^26^	27.08	>15 minutes	Postoperative Until 7 days	POAF= 20.8 ± 6.9%, no POAF = 30 ± 12.8%	–	–	POAF= 11.1 ± 3.8%, no POAF = 14.6 ± 7.0%	POAF = 41.1 ± 9.2, no POAF = 32.6 ± 9.0 Cut-off LAVi >36	GE EchoPAC	Vivid 7, GE Vingmed Ultrasound AS
10	Başaran et al (2016)^27^	25.56	>15 minutes	Postoperative Until 4 days	POAF = 24.2 ± 5.8, no POAF= 31.7 ± 9.6,	–	–	–	POAF = 34 ± 11.3, no POAF = 26.4 ± 8.4	GE EchoPAC	Vivid 7, GE Vingmed Ultrasound AS
11	Pernigo et al (2017)^28^	43.33	>30 seconds	Postoperative Until 3 days	POAF = 18.1 + 5.3; no POAF = 27.1 + 6.7	23	POAF = 8.4 ± 3.1, no POAF = 14.3 ± 4.7	–	POAF = 42.5 ± 14.3, no POAF = 37.2 ± 9.8	Philips QLAB	Philips HD15 or iE33 ultrasound system
12	Aksu et al (2017)^29^	31.67	–	–	POAF = 34.6 ± 3.5; no POAF= 36.1 ± 4.3	–	–	–	POAF = 42 ± 9 Cut-off >46	EchoPAC	GE Vivid 7
13	Pessoa et al (2018)^30^	31.58	New onset AF	Postoperative Until discharge	–	18.7	Cut-off value < 7.9%	–	Cut-off LA vol >68 mL	SyngoVVI, Siemens	Philips iE33 ultrasound system
14	Lisi et al (2018)^31^	32.43	–	Postoperative Until 3 months Early POAF (2.9 + 1.9 days)	POAF = 18.1 ± 5.4, no POAF = 28.3 ± 6.3	–	POAF = 13.2 ± 4, no POAF = 12.6 ± 4.2	–	POAF66.9 ± 25.5, no POAF = 45.1 ± 16.1	GE EchoPAC	GE Vivid 7
15	Sabry et al (2020)^32^	22	≥30 seconds	Postoperative Until discharge and till 1 month	POAF = 20.4 + 1.7; no POAF = 22.1 + 1.9	23.1	–	–	–	Phillips software	Philips iE33 xMatrix—DS
16	Rizvi et al (2020)^33^	37.78	>30 seconds	Postoperative Until discharge	POAF = 6.9 ± 0.69; no POAF = 10.9 ± 0.93	–	–	–	–	Toshiba Aplio Artida with 3 D strain capability	Toshiba Aplio Artida
17	Darweesh et al (2021)^34^	28.57	≥30 seconds	Postoperative Until discharge	POAF = 30.30 + 4.95, no POAF = 46.91 + 5.76	–	POAF 23.93 + 4.19, no POAF = 37.00 ± 3.35	POAF = 27.15 + 4.09, no POAF = 32.15 ± 4.20	POAF = 32.22 + 7.13, no POAF= 26.87 ± 6.98	QLabsoftware (cardiac motion quantification (CMQ); Phillips Medical Systems)	Philips EPIQ 7 unit
18	Abdelrazek et al (2021)^35^	31.46	>30 seconds	Postoperative Until 7 days	POAF = 25.6 ± 6.1, no POAF = 32.9 ± 5.9 POAF = 1.27 ± 0.56 per second, no POAF = 1.90 ± 0.71 per second	29.8	POAF= −1.21 ± 0.48 per second, no POAF= −2.21 ± 0.59 per second	POAF= −1.0 ± 0.45 per second, no POAF= −1.69 ± 0.6 per second	–	Philips Q lab	Philips iE33
19	Kislitsina et al (2022)^36^	23.69	>15 minutes	Postoperative Until 3 days	POAF = 22.9 + 8.3 (n = 47); no POAF = 32.8 + 11.2 (n = 47)	–	POAF = 11.4 ± 5.3, no POAF = 18.5 ± 6.7	–	POAF = 29.8 ± 10.5, no POAF = 24.9 ± 9.9	TomTec	TomTec
20	Dalos et al (2022)^37^	43.54	–	Postoperative Until 9 (7-12) days	POAF = 14.50 + 5.26; no POAF= 19.23 + 6.59	17	–	–	POAF = 44.5(37.2-55.3), no POAF = 42.2(32.9-53.4)	GE EchoPAC	GE Vivid E9 and E95 scanners
21	Wedin et al (2024)^38^	50.2	>30 seconds	Postoperative up to 3 days	–	24.4	–	–	–	TomTec CPA	Tom Tec
22	Pastore et al (2024)^39^	33.23	>30 seconds	Postoperative until discharge	POAF = 22 + 7.9; no POAF = 29.7 + 9.7	28	POAF = 13 + 5.4, no POAF= 15 ± 6.5	–	POAF = 31.5 + 11, no POAF = 31 ± 10	TomTec CPA; GE EchoPAC	EpiQ, Philips Healthcare, GE Vivid E9
23	Borde et al (2024)^40^	23.30	New onset AF	Postoperative up to 3 days	POAF = 19.2 ± 4.7%, no POAF = 23.5 ± 4.8%	23	POAF = 10.3 ± 3.9, no POAF = 12.1 ± 4.1	POAF = 8.9 ± 3.7, no POAF = 12.3 ± 4.8	POAF = 26 ± 9, no POAF = 24 ± 7.3	GE EchoPAC	GE Vivid T8
24	Granchietti et al (2025)^41^	27	New onset AF	Postoperative up to 3.2 days	POAF = 18.8 ± 9.4%, no POAF = 25.2 ± 10.4%	24.5	POAF = 8.7 ± 6.8, no POAF = 12.3 ± 7.6 Cut-off <9.5	POAF = 10.1 ± 6.1, no POAF = 13.1 ± 7.1	POAF = 36.2 ± 11.8, no POAF = 31.6 ± 9.7	Acoustic tracking software	Aplio i900 ultrasound machine (Canon Medical Systems Corporation, Tokyo, Japan)

Abbreviations: LA, left atrium; POAF, postoperative atrial fibrillation; SD, standard deviation.

Eleven studies used GE EchoPAC,^19–21^^,^^24–27^^,^^29^^,^^31^^,^^37^^,^^40^ whereas 3 studies used Philips QLAB^28^^,^^34^^,^^35^ for LA strain analysis. Remaining studies used various other softwares, such as Syngo VVI,^18^^,^^30^ Toshiba Ultra Extend,^22^ Xstraine licence software,^23^ Philips software (not specified),^32^ Toshiba Aplio Artida with 3D strain capability,^33^ TomTec,^36^ TomTec CPA,^38^ acoustic tracking software,^41^ or both TomTec CPA and GE EchoPAC^39^ for LA strain analysis.

### Quality of the studies

As per the Newcastle Ottawa scale,^16^ 19 studies^18–28^^,^^31–35^^,^^37^^,^^39^^,^^41^ were graded as very good with a score of 9, 3 studies^29^^,^^36^^,^^40^ were considered good with a score of 8, whereas 2 studies^30^^,^^38^ were satisfactory with score of 6. Reasons for downgrading the studies to satisfactory were absence of data regarding selection and comparability of cohorts or analysis uncontrolled for confounders. No studies were deemed to be unsatisfactory (**Table S2**).

### GRADE

The certainty of evidence for the primary objective of LA reservoir strain was downgraded to low due to imprecision of the results as assessed by wide 95% CI for most studies and inconsistency since heterogeneity was high (**Table S3**).

### Study outcomes

**Figure 2.**
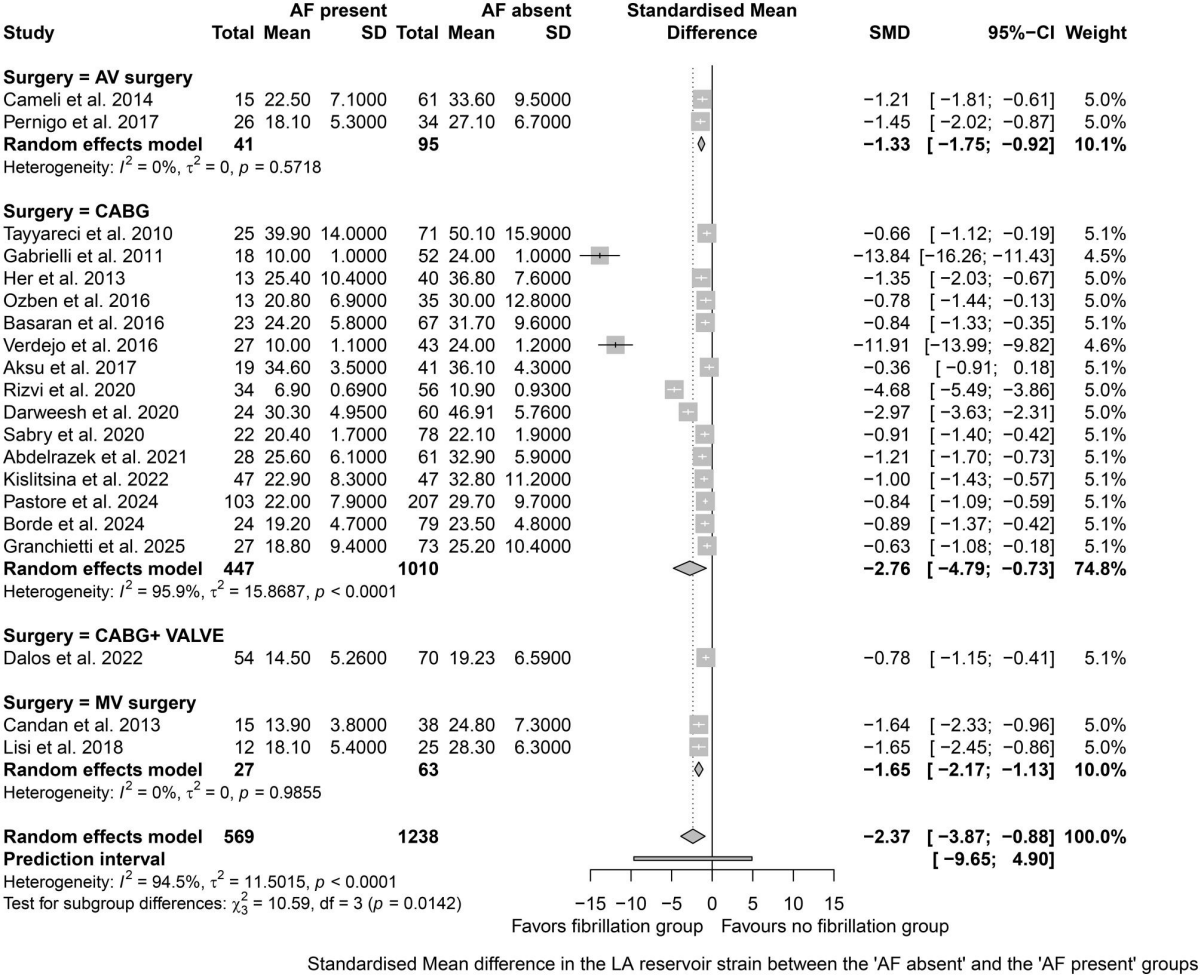
Forest Plot Depicting the Meta-Analysis of LA Reservoir Strain Based on Type of Surgery (POAF vs no POAF: SMD −2.37; 95% CI, −3.87 to −0.88; Prediction Interval: −9.60 to 4.90; *I*^2^ = 94.5%). Abbreviations: AF, atrial fibrillation; AV surgery, aortic valve surgery; CABG, coronary artery bypass graft; CI, confidence interval; LA, left atrium; MV surgery, mitral valve surgery; POAF, postoperative atrial fibrillation; SMD, standardized mean difference.

**Figure 3.**
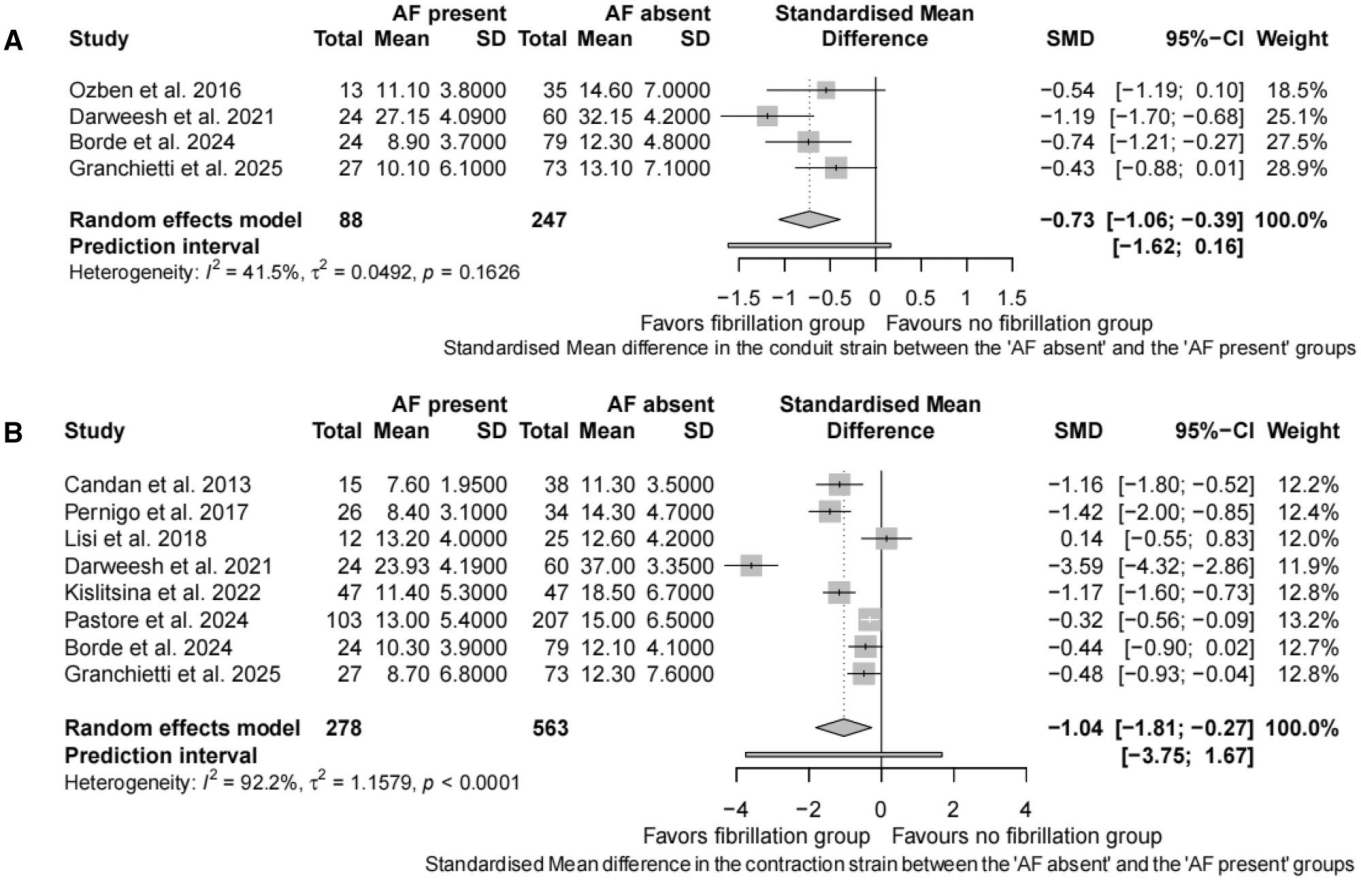
Forest Plot Depicting the Meta-Analysis of : (A) LA Conduit Strain (POAF vs no POAF: SMD −0.73; 95% CI, −1.06 to −0.39; Prediction Interval: −1.62 to −0.16; *I*^2^ = 41.5%) and (B) Contraction Strain (POAF vs no POAF: SMD −1.04; 95% CI, −1.81 to −0.27; Prediction Interval: −18.33 to 11.32; *I*^2^ = 92.2%). Abbreviations: AF, atrial fibrillation; CI, confidence interval; LA, left atrium; POAF, postoperative atrial fibrillation; SMD, standardized mean difference.

**Figure 4.**
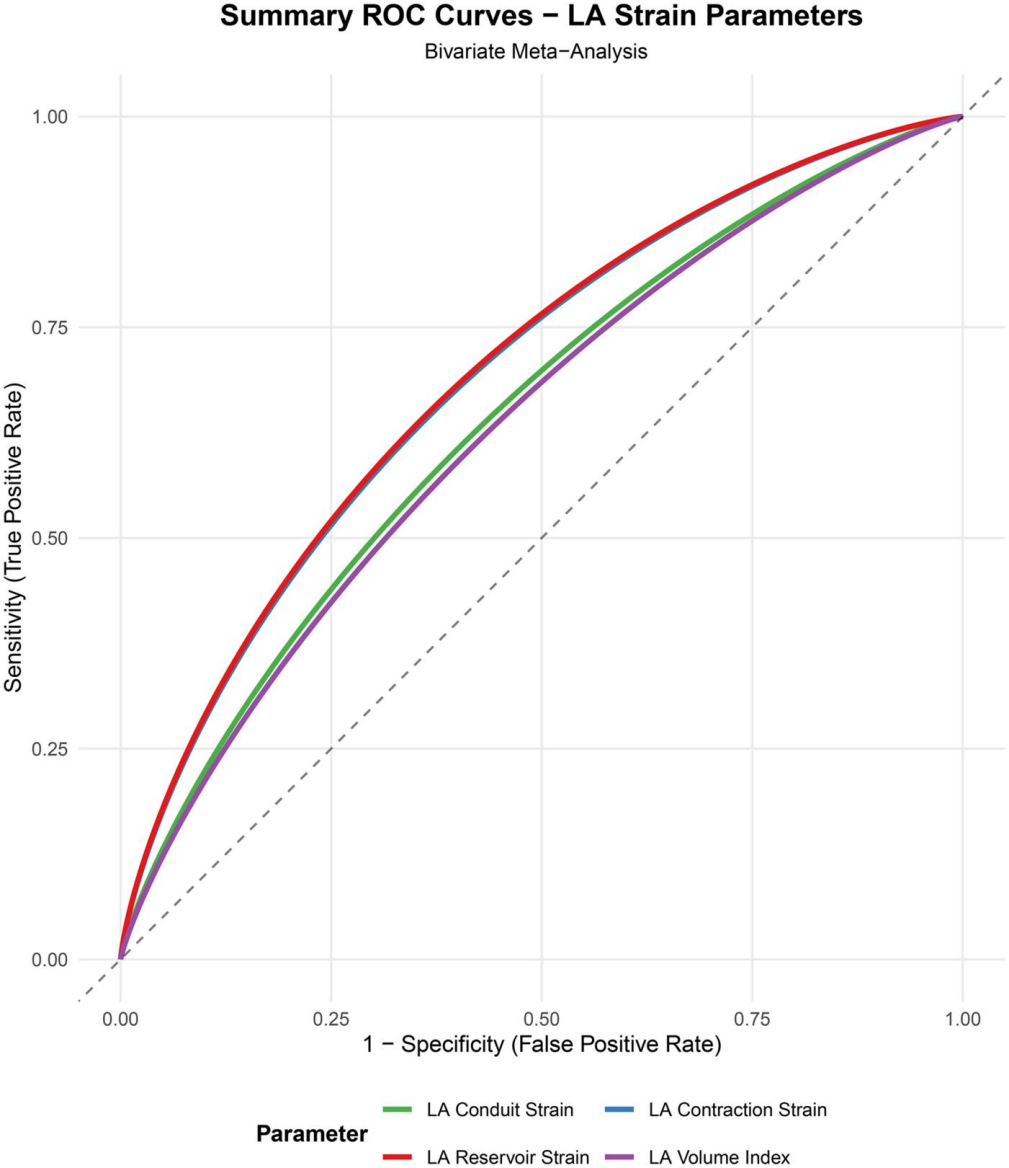
Bivariate ROC Analysis Recommending Cut-Off of LA Reservoir Strain 22% to 25%, Conduit Strain 12%, Contraction Strain 12% to 14%, and LAVI 34 mL/m^2^ for Predicting POAF. Abbreviations: LA, left atrium; LAVI, left atrial volume index; POAF, postoperative atrial fibrillation; ROC, receptor operating characteristics.

- Meta-analysis for preoperative LA reservoir strain:
  Twenty studies were included for meta-analysis of preoperative LA reservoir strain (**Figure 2**).^18–21^^,^^24–29^^,^^31–37^^,^^39–41^ Preoperative LA reservoir strain was significantly lower in patients with POAF vs those without POAF (SMD −2.37; 95% CI, −3.87 to −0.88). The 95% prediction interval for LA reservoir strain was −9.65 to 4.9, suggesting that the true effect size might vary in future studies. Based on incidence of POAF, 1 study reported an incidence <20% (SMD −1.56; 95% CI, −2.37 to −0.75), while 11 studies reported incidence of 20%-30% (SMD −1.66; 95% CI, −2.42 to −0.90), and 8 studies reported incidence >30% (SMD −2.96; 95% CI, −5.53 to −0.39).
  A significant heterogeneity was noted for LA reservoir strain (*I*^2^ = 94.5%). Leave-one-out sensitivity analysis did not change either the heterogeneity or the direction of the result. Subgroup analysis was performed based on the type of surgery, vendor specific platform for LA strain analysis, and according to incidence of POAF reported. Heterogeneity was noted mainly for CABG surgeries (*I*^2^ = 95.9%) but not for valve surgeries. Significant heterogeneity was noted across all vendor platforms contributing to the overall heterogeneity (GE: *I*^2^ = 95.6%; Phillips: *I*^2^ =88.4%; others: *I*^2^ = 95.2%). Multivariate meta regression for heterogeneity in reservoir strain was significant for gender, vendor platform, and filling pressures (E/e’) (**Table S4**).
- Meta-analysis for preoperative LA conduit strain:
  Four studies were included for meta-analysis of preoperative LA conduit strain (**Figure 3A**).^26^^,^^34^^,^^40^^,^^41^ Preoperative LA conduit strain was significantly lower in patients with POAF vs those without POAF (SMD −0.73; 95% CI, −1.06 to −0.39). The 95% prediction interval for LA conduit strain was −1.62 to 0.16, thus suggesting that the values of conduit strain can be extrapolated to future studies. The heterogeneity of the model was acceptable at *I*^2^ = 41.5%.
- Meta-analysis for preoperative LA contraction strain:
  Eight studies were included for meta-analysis of preoperative LA contraction strain (**Figure 3B**).^21^^,^^28^^,^^31^^,^^34^^,^^36^^,^^39–41^ Preoperative LA contraction strain was significantly lower in patients with POAF vs those without POAF (SMD −1.04; 95% CI, −1.81 to −0.27). The 95% prediction interval for LA contraction strain was −3.75 to 1.67, thus precluding its extrapolation to future cohort of patients.
  A significant heterogeneity was noted for LA contraction strain (*I*^2^ = 92.2%) which was further quantified by sensitivity analysis, but it did not change either the heterogeneity or direction of the result.
- Meta-analysis for preoperative LA strain rate:
  Seven studies were synthesized for preoperative LA reservoir strain rate,^18–20^^,^^22^^,^^23^^,^^25^^,^^35^ 5 for conduit strain rate,^18^^,^^20^^,^^22^^,^^23^^,^^35^ and 6 for contraction strain rate.^19^^,^^21–23^^,^^25^^,^^35^ Preoperative LA reservoir strain rate was not different in patients with POAF vs those without POAF (SMD −9.36; 95% CI, −24.95 to 6.24) (**Figure S1A**). The 95% prediction interval for LA reservoir strain rate was −64.23 to 45.52. The heterogeneity of the model was high (*I*^2^ = 97.3%) which was further quantified by sensitivity analysis, but it did not change either the heterogeneity or the direction of the results.
  Preoperative LA conduit strain rate was not different in patients with POAF vs those without POAF (SMD: 0.27; 95% CI, −0.58 to 1.12) (**Figure S1B**). The 95% prediction interval for LA conduit strain rate was −2.57 to 3.11. The heterogeneity of the model was high (*I*^2^ = 91.6%). Sensitivity analysis did not change either the heterogeneity or the direction of the results.
  Preoperative LA contraction strain rate was also not different in patients with POAF vs those without POAF (SMD: −3.51; 95% CI, −7.8 to 0.79) (**Figure S1C**). The 95% prediction interval for LA contraction strain rate was −18.33 to 11.32. The heterogeneity of the model was high (*I*^2^ = 98.4%). Sensitivity analysis did not change either the heterogeneity or the direction of the results.
- Meta-analysis for preoperative LAVI:
  Seventeen studies were included for meta-analysis of preoperative LAVI (**Figure S2**).^17–23^^,^^25–27^^,^^30^^,^^33^^,^^35^^,^^36^^,^^38–40^ Preoperative LAVI was significantly higher in patients with POAF vs those without POAF (SMD 0.7; 95% CI, 0.33 to 1.08). The 95% prediction interval for LAVI was −0.91 to 2.32, thus precluding its extrapolation to future cohort of patients. The heterogeneity of the model was high at *I*^2^ = 86.9% which was further quantified by sensitivity analysis, but it did not change either the heterogeneity or the direction of the results.
- Cut-off values for predicting POAF:
  Bivariate ROC analysis showed a cut-off value of LA reservoir strain 22%-25% as having the highest AUC among all the parameters (**Figure 4**, **Table 3**). In patients undergoing CABG surgery, a cut-off of 21.5% (AUC 0.76, sensitivity 59.9%, specificity 88.2%) was obtained using the GE EchoPAC.
- Publication bias
  Publication bias was evaluated for all studies reporting reservoir strain since there were insufficient studies (< 10) for conduit and contraction strain. No significant publication bias was noted for reservoir strain (*P* = .72; **Figure S3**).

**Table 3.** Summary of Bivariate ROC Analysis and Cut-Offs for POAF

Parameter	Recommended cut-off	Sensitivity (95% CI)	Specificity (95% CI)	AUC
LA reservoir strain	22%-25%	0.713 (0.675-0.743)	0.679 (0.645-0.711)	0.696
LA conduit strain	12%	0.634 (0.554-0.715)	0.653 (0.566-0.731)	0.644
LA contraction strain	12%-14%	0.628 (0.548-0.786)	0.752 (0.582-0.869)	0.690
LA volume index	34 mL/m^2^	0.629 (0.584-0.679)	0.638 (0.585-0.687)	0.633

Abbreviations: AUC, area under curve; CI, confidence interval; LA, left atrium; POAF, postoperative atrial fibrillation; ROC, receptor operating characteristics.

## DISCUSSION

The main findings of the current meta-analysis were that low preoperative LA strain parameters (reservoir, conduit, and contraction strain) but not strain rates predispose patients to POAF following cardiac surgery. In this regard, the best cut-off values for predicting POAF was found for LA reservoir strain.

There is considerable lack of consensus in defining POAF.^43^ As per the American Association of Thoracic surgery 2014 guidelines, POAF is defined as either AF lasting > 30 seconds and/or requiring treatment, anticoagulation, or extending the duration of hospital stay following thoracic surgery.^44^ Other guidelines provide varied definitions of AF but not specific to POAF following cardiac surgery.^45–47^

POAF incidence varies according to the surgery, with MV surgeries conferring a higher risk of approximately 40%,^2^ whereas incidence following CABG and AV surgeries are ∼ 26% and 30%, respectively.^3^ An estimated 90% to 98% of new-onset POAF resolve within 4 weeks because of LA reverse remodelling occurring predominantly in the first 30 days after surgery.^44^ Therefore, in this review, we included all studies reporting POAF until 30 days of index surgery.

Recent evidence has focused on the LA for outcome prediction models following cardiac surgery, since the structure and function of the thin-walled atria may be impacted earlier than the ventricles in patients with cardiovascular diseases.^48^ In this regard, LA strain parameters capture the atrial function and have been found to deteriorate earlier than structural changes such as increases in atrial volumes.^12^^,^^49^ Thus, utilizing strain values may be superior to other measures of LA function such as chamber size, area, or volume. With current evolutions in technology, LA strain specific software with 2D STE is now available on most echocardiography platforms where the region of interest can be mapped more accurately on to the thin walled LA myocardium.^48^^,^^50^

The relationship between low LA strain values and increased incidence of POAF may be explained due to oxidative stress and inflammation leading to atrial fibrosis, which causes impairment of STE parameters and may provoke re-entry circuits triggering AF under an external stimulus, such as cardiac surgery. Over this underlying substrate, surgery-related atrial manipulation, cardiopulmonary bypass, and ischaemia-reperfusion mechanisms further increase inflammation and oxygen radical formation triggering the arrhythmogenic activity in the background of pre-existing LA structural abnormalities. The structural abnormalities may evolve differently in patients undergoing valve surgeries vs those undergoing CABG.^6^^,^^7^ In CABG, diastolic dysfunction is common, and independently associated with POAF development,^51^ whereas in patients with valvular heart disease, the haemodynamic burden causes progressive LA enlargement which provides the arrhythmogenic substrate for subsequent development of POAF.^6^^,^^7^^,^^13^

While LA reservoir strain indicates the compliance of the LA, the reservoir strain rate implies the rate of change of deformation of LA in systole which is influenced by both elasticity as well as compliance of the atrial wall.^18^ In our review, reservoir strain rate did not predict POAF. This may suggest that the rate of deformation may decline only with deterioration in both compliance as well as elasticity. In addition, strain rate parameters depend on heart rate, and image quality, making these measurements not commonly practiced in perioperative period.

A previous meta-analysis^13^ published in 2021 examined the role of pre-operative transthoracic echocardiography in predicting POAF after cardiac surgery. The findings of the previous review^13^ for LA reservoir strain are consistent with our findings. However, the previous review^13^ included only 5 studies for LA reservoir strain,^21^^,^^24^^,^^27^^,^^28^^,^^31^ of which 4^21^^,^^24^^,^^28^^,^^31^ were in cardiac valvular surgeries, whereas we have synthesized a wide spectrum of cardiac surgeries for LA reservoir strain, including CABG and valvular surgeries, thereby increasing the generalizability of our findings. Other shortcomings of the previous meta-analysis which were addressed in our review is the inclusion of all LA strain and strain rate parameters, cut-off values of LA strain parameters, robust meta-regression, plus subgroup analysis to explore heterogeneity. Furthermore, prediction intervals and cut-offs were not provided in the previous meta-analysis,^13^ which would be useful to the clinician when designing and interpreting future studies on LA strain. Finally, the overall effect size for LA reservoir strain (SMD 2.37) in our study is more than the previous review (SMD 1.4),^13^ thus improving the strength of the findings.

Another systematic review on the prognostic value of strain and strain rate in the prediction of POAF was carried out only in patients undergoing CABG,^52^ which makes it impossible to extrapolate the information in patients undergoing other types of cardiac surgeries. Furthermore, the review included 6 studies, with LA strain being measured by STE in only 2 studies.^19^^,^^20^ A third meta-analysis of 12 studies in patients undergoing CABG was reported only as an abstract.^53^

The population normal reference range for reservoir, conduit, and contraction strain are 39%-42.5%, 23%-25%, and 16%-17%, respectively.^54^^,^^55^ In our meta-analysis, we found the cut-offs of 22%-25%, 12%, and 12%-14%, respectively, for reservoir, conduit, and contraction strain in predicting POAF. For CABG surgery only using the GE EchoPAC software, the cut-off for reservoir strain was 21.5%. This implies that a substantial decrease in LA function must occur before predisposing patients to POAF. Since this review found the widest SMDs (2.96) with a higher incidence of POAF (>30%), the applications of LA strain parameters to predict POAF may be more accurate where the incidence of POAF is high; for example, in patients with risk factors for developing POAF who would ultimately benefit from a targeted approach.

Despite our rigorous and comprehensive approach, this systematic review and updated meta-analysis has some limitations that warrant consideration. Inclusion of only observational studies further limits the strength of our findings. Strain being vendor specific makes it difficult to interpret pooled data. Patient-level data were not analysed for certain analysis such as predicting the cut-off values, resulting in loss of information and signals because of averaging. No studies reported outcomes following POAF, and thus, it is unknown whether POAF conferred morbidity and/or mortality in patients after cardiac surgery in the included studies. Our sensitivity and specificity for LA reservoir strain was modest, and likely due to the observational nature of the studies. Finally, the results of our meta-regression and subgroup analysis indicate potential co-variates which require further consideration during interpretation of results and the design of future studies.

## CONCLUSION

This meta-analysis provides convincing evidence that decreased preoperative LA strain parameters predispose adult patients undergoing cardiac surgery to POAF. The largest pool of evidence is regarding the LA reservoir strain. Implications for practice should be to include the LA reservoir strain as a preoperative risk tool for predicting POAF given the ease of LA strain analysis by newer dedicated softwares.^56^ Implications for research should be to design randomized controlled trials (RCTs) that compare outcomes following POAF using strain analysis.

## Supplementary Material

ivag035_Supplementary_Data

## Data Availability

All data are included in the manuscript
