# Supplementary material for: Predictive Value of Preoperative Left Atrial Strain Parameters on Postoperative Atrial Fibrillation in Adults Undergoing Cardiac Surgery: A Systematic Review and Meta-Analysis
Source: Interdiscip Cardiovasc Thorac Surg. 2026 Feb 13;41(2):ivag035. doi: 10.1093/icvts/ivag035 (PMC12920041; doi:10.1093/icvts/ivag035)
Supplement: ivag035_Supplementary_Data [file ivag035_supplementary_data.zip › Supplementary figure legends.docx]

**Supplementary figure legends**

Supplementary figure 1: Forest plot showing the meta-analysis of (a) LA reservoir strain rate (SMD -9.36; 95% CI: -24.95 to 6.24), (b) conduit strain rate (SMD: 0.27; 95% CI: -0.58 to 1.12), and (c) contraction strain rate (SMD: -3.51; 95% CI: -7.8 to 0.79). AF, atrial fibrillation; LA, left atrium.

Supplementary figure 2: Forest plot depicting the meta-analysis of LAVI based on type of surgery (POAF vs. no POAF: SMD 0.7; 95% CI: 0.33 to 1.08; prediction interval -0.91 to 2.32). POAF, postoperative atrial fibrillation; CI, confidence interval; LA, left atrium; LAVI, left atrial volume index; SMD, standardized mean difference.

Supplementary figure 3: Funnel plot for publication bias of included studies for LA reservoir strain suggests an absence of publication bias. LA, left atrium
