# Supplementary material for: Predictive Value of Preoperative Left Atrial Strain Parameters on Postoperative Atrial Fibrillation in Adults Undergoing Cardiac Surgery: A Systematic Review and Meta-Analysis
Source: Interdiscip Cardiovasc Thorac Surg. 2026 Feb 13;41(2):ivag035. doi: 10.1093/icvts/ivag035 (PMC12920041; doi:10.1093/icvts/ivag035)
Supplement: ivag035_Supplementary_Data [file ivag035_supplementary_data.zip › Supplementary table 1.docx]

**Supplementary table 1:** Detailed search strategy

| *Search entry PubMed* | | |
| --- | --- | --- |
| MeSH | Medical subject headings |  |
| tiab | Words in title or abstract |  |
| * | Truncation |  |
| Search | Entry terms | Result |
| #1 | Left atrial strain | [46](https://pubmed.ncbi.nlm.nih.gov/?term=%28left+atrial+strain%29&sort=)61 |
| #2 | Left atrial reservoir strain | 1359 |
| #3 | #1 OR #2 | 4661 |
| #4 | atrial fibrillation [MeSH Terms] | 126434 |
| #5 | atrial fibrillation[tiab] OR atrial fibr*[tiab] OR postoperative atrial fibrillation[tiab] | 111127 |
| #6 | #4 OR #5 | 126804 |
| #7 | #3 AND #6 | 1419 |
| #8 | cardiac surgical procedures [MeSH Terms] | 259452 |
| #9 | cardiac surgical procedures [Title/Abstract] OR cardiac surgical proc*[Title/Abstract] | 2566 |
| #10 | thoracic surgery [MeSH Terms] OR cardiac surgical procedures [MeSH Terms] | 400890 |
| #11 | cardiac surgery [Title/Abstract] OR cardiac surg*[Title/Abstract] | 64198 |
| #12 | sternotomy [MeSH Terms] | 3384 |
| #13 | sternotomy [Title/Abstract] | [127](https://pubmed.ncbi.nlm.nih.gov/?term=sternotomy%5BTitle%2FAbstract%5D&sort=)22 |
| #14 | #8 OR #9 OR #10 OR #11 OR #12 OR #13 | 433401 |
| #15 | #7 AND #14 | 88 |
| *Search entry Embase* | | |
| exp | Emtree keyword with explosion |  |
| ab,ti | Words in title or abstract |  |
| * | Truncation |  |
| Search | Entry terms | Result |
| #1 | Left AND atrial AND strain | 10869 |
| #2 | Left AND atrial AND reservoir AND strain | 3050 |
| #3 | #1 OR #2 | 10869 |
| #4 | Atrial AND fibrillation | 285818 |
| #5 | Postoperative atrial fibrillation | 3663 |
| #6 | #4 OR #5 | 285818 |
| #7 | #3 AND #6 | 4302 |
| #8 | Heart AND surgery | 848006 |
| #9 | Thorax AND surgery | 236834 |
| #10 | #8 OR #9 | 1003882 |
| #11 | #7 AND #10 | 778 |
| *Search entry Cochrane* | | |
| MeSH | Medical subject headings |  |
| * | Truncation |  |
| Search | Entry terms | Result |
| #1 | atrial fibrillation | 18282 |
| #2 | postoperative atrial fibrillation | 2088 |
| #3 | #1 OR #2 | 18282 |
| #4 | Left atrial strain | 409 |
| #5 | Left atrial reservoir strain | 73 |
| #6 | #4 OR #5 | 409 |
| #7 | #3 AND #6 | 169 |
| #8 | thoracic surgery | 16037 |
| #9 | cardiac surgical procedures | 6077 |
| #10 | cardiac surg* cardiac surgical procedures OR sternotom* | 7481 |
| #11 | #8 OR #9 OR #10 | 22033 |
| #12 | #7 AND #10 | 4 |
| *Search entry Google Scholar* | | |
|  | *left atrial strain and postoperative atrial fibrillation and cardiac surgery* | 2 |
| Summary | | |
| Combined search results from all search engines | | 872 |
|  | |  |
