# Supplementary material for: Predictive Value of Preoperative Left Atrial Strain Parameters on Postoperative Atrial Fibrillation in Adults Undergoing Cardiac Surgery: A Systematic Review and Meta-Analysis
Source: Interdiscip Cardiovasc Thorac Surg. 2026 Feb 13;41(2):ivag035. doi: 10.1093/icvts/ivag035 (PMC12920041; doi:10.1093/icvts/ivag035)
Supplement: ivag035_Supplementary_Data [file ivag035_supplementary_data.zip › Supplementary table 2.docx]

**Supplementary table 2:** Newcastle Ottawa scale

|  |  | **Selection** | | | | **Comparability** | **Outcome** | | |  |
| --- | --- | --- | --- | --- | --- | --- | --- | --- | --- | --- |
| Sl no. | Study | Representativeness of the exposed cohort | Selection of the non-exposed cohort | Ascertainment of exposure | Demonstration that outcome of interest was not present at start of study | Comparability of cohorts on the basis of the design or analysis controlled for confounders | Assessment of outcome | Was follow-up long enough for outcomes to occur | Adequacy of follow-up of cohorts | Total |
| 1 | Tayyareci et al. 2010 | 1 | 1 | 1 | 1 | 2 | 1 | 1 | 1 | 9 |
| 2 | Gabrielli et al. 2011 | 1 | 1 | 1 | 1 | 2 | 1 | 1 | 1 | 9 |
| 3 | Her et al. 2013 | 1 | 1 | 1 | 1 | 2 | 1 | 1 | 1 | 9 |
| 4 | Candan et al. 2013 | 1 | 1 | 1 | 1 | 2 | 1 | 1 | 1 | 9 |
| 5 | Imanishi et al. 2014 | 1 | 1 | 1 | 1 | 2 | 1 | 1 | 1 | 9 |
| 6 | Parsaee et al. 2014 | 1 | 1 | 1 | 1 | 2 | 1 | 1 | 1 | 9 |
| 7 | Cameli et al. 2014 | 1 | 1 | 1 | 1 | 2 | 1 | 1 | 1 | 9 |
| 8 | Verdejo et al. 2016 | 1 | 1 | 1 | 1 | 2 | 1 | 1 | 1 | 9 |
| 9 | Ozben et al. 2016 | 1 | 1 | 1 | 1 | 2 | 1 | 1 | 1 | 9 |
| 10 | Başaran et al. 2016 | 1 | 1 | 1 | 1 | 2 | 1 | 1 | 1 | 9 |
| 11 | Pernigo et al. 2017 | 1 | 1 | 1 | 1 | 2 | 1 | 1 | 1 | 9 |
| 12 | Aksu et al. 2017 | 1 | 1 | 1 | 1 | 2 | 1 | 1 | 0 | 8 |
| 13 | Pessoa et al*.* 2018 | 1 | 0 | 1 | 1 | 0 | 1 | 1 | 1 | 6 |
| 14 | Lisi et al. 2018 | 1 | 1 | 1 | 1 | 2 | 1 | 1 | 1 | 9 |
| 15 | Sabry et al. 2020 | 1 | 1 | 1 | 1 | 2 | 1 | 1 | 1 | 9 |
| 16 | Rizvi et al*.* 2020 | 1 | 1 | 1 | 1 | 2 | 1 | 1 | 1 | 9 |
| 17 | Darweesh et al. 2021 | 1 | 1 | 1 | 1 | 2 | 1 | 1 | 1 | 9 |
| 18 | Abdelrazek et al. 2021 | 1 | 1 | 1 | 1 | 2 | 1 | 1 | 1 | 9 |
| 19 | Kislitsina et al. 2022 | 1 | 1 | 1 | 0 | 2 | 1 | 1 | 1 | 8 |
| 20 | Dalos et al. 2022 | 1 | 1 | 1 | 1 | 2 | 1 | 1 | 1 | 9 |
| 21 | Wedin et al. 2024 | 1 | 0 | 1 | 1 | 0 | 1 | 1 | 1 | 6 |
| 22 | Pastore et al. 2024 | 1 | 1 | 1 | 1 | 2 | 1 | 1 | 1 | 9 |
| 23 | Borde et al. 2024 | 1 | 1 | 1 | 0 | 2 | 1 | 1 | 1 | 8 |
| 24 | Granchietti et al. 2025 | 1 | 1 | 1 | 1 | 2 | 1 | 1 | 1 | 9 |

**Grading of studies:**

1. Cross-sectional studies: Very good= 6-7; Good= 5; Satisfactory= 4; Unsatisfactory= 0-3
2. Cohort studies: Very good= 9-10; Good=7-8; Satisfactory= 5-6; Unsatisfactory= 0-4
