# Supplementary material for: Predictive Value of Preoperative Left Atrial Strain Parameters on Postoperative Atrial Fibrillation in Adults Undergoing Cardiac Surgery: A Systematic Review and Meta-Analysis
Source: Interdiscip Cardiovasc Thorac Surg. 2026 Feb 13;41(2):ivag035. doi: 10.1093/icvts/ivag035 (PMC12920041; doi:10.1093/icvts/ivag035)
Supplement: ivag035_Supplementary_Data [file ivag035_supplementary_data.zip › Supplementary table 3.docx]

| No. of studies | Certainty assessment | | | | | |  | | |
| --- | --- | --- | --- | --- | --- | --- | --- | --- | --- |
|  | Study design | Risk of bias | Inconsistency | Indirectness | Imprecision | Other considerations | Effect  (POAF vs no POAF) | Certainty | Importance |
| 20 | Observational | Not Serious | Serious^a^ | Not serious | Serious^b^ | Not serious | SMD: -2.37%; 95% CI -3.87 to -0.88 | ⨁◯◯◯  Low | Critical |

**Supplementary table 3:** GRADE Table for certainty of evidence (Outcome assessed: LA reservoir strain)

1. We downgraded the evidence since heterogeneity is high.
2. We downgraded the evidence since the 95% CI for most studies were wide.

Abbreviations: LA, left atrium; SMD, standardized mean difference; CI, confidence interval; POAF, postoperative atrial fibrillation
