# Supplementary material for: Predictive Value of Preoperative Left Atrial Strain Parameters on Postoperative Atrial Fibrillation in Adults Undergoing Cardiac Surgery: A Systematic Review and Meta-Analysis
Source: Interdiscip Cardiovasc Thorac Surg. 2026 Feb 13;41(2):ivag035. doi: 10.1093/icvts/ivag035 (PMC12920041; doi:10.1093/icvts/ivag035)
Supplement: ivag035_Supplementary_Data [file ivag035_supplementary_data.zip › Supplementary table 4.docx]

**Table 4:** Meta regression of LA reservoir strain*

| **Rank** | **Variable** | **I² Reduction** | **% Reduction** | **P-value** | **Significance** |
| --- | --- | --- | --- | --- | --- |
| **1** | **LV GLS Difference** | **12.35%** | 12.47% | 0.656 | Not Significant |
| **2** | **LAVI Difference** | **11.42%** | 11.53% | 0.298 | Not Significant |
| **3** | **Average E/e' Difference** | **8.04%** | 8.12% | **0.072** | **Significant** |
| **4** | Male % Difference | 5.72% | 5.78% | **0.080** | **Significant** |
| 5 | LVEF Difference | 3.87% | 3.91% | 0.766 | Not Significant |
| 6 | Age Difference | 2.60% | 2.63% | 0.342 | Not Significant |
| 7 | Vendor | 1.00% | 1.01% | **0.099** | **Significant** |
| 8 | POAF Definition | 0.34% | 0.34% | 0.848 | Not Significant |
| 9 | Sample Size | -0.01% | -0.01% | 0.303 | Not Significant |
| 10 | Surgery Type | -0.16% | -0.17% | 0.429 | Not Significant |

*We used P <0.10 for significance in the meta-regression because of the exploratory nature. Further, the purpose of this meta-regression is to explain the heterogeneity and not to test any hypothesis. Moreover, the number of studies included are relatively less. So, using P<0.05 will be overly conservative.
