# Supplementary figures and images for: Predictive Value of Preoperative Left Atrial Strain Parameters on Postoperative Atrial Fibrillation in Adults Undergoing Cardiac Surgery: A Systematic Review and Meta-Analysis
Source: Interdiscip Cardiovasc Thorac Surg. 2026 Feb 13;41(2):ivag035. doi: 10.1093/icvts/ivag035 (PMC12920041; doi:10.1093/icvts/ivag035)

(a)

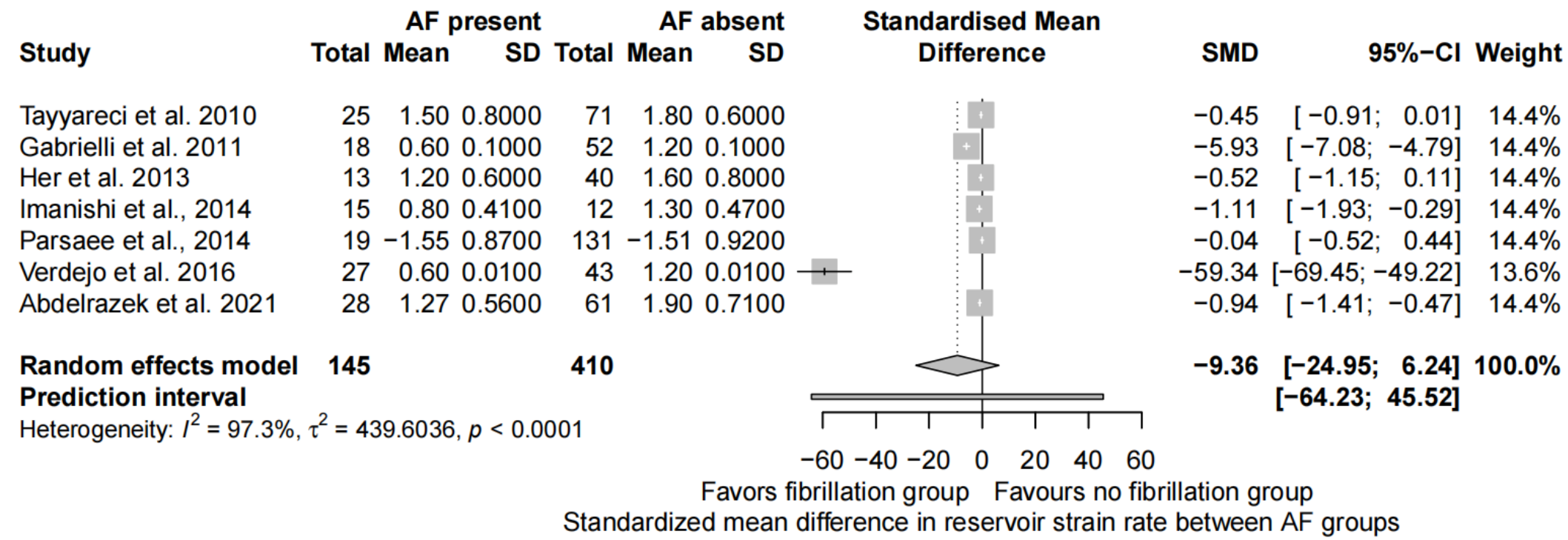

(b)

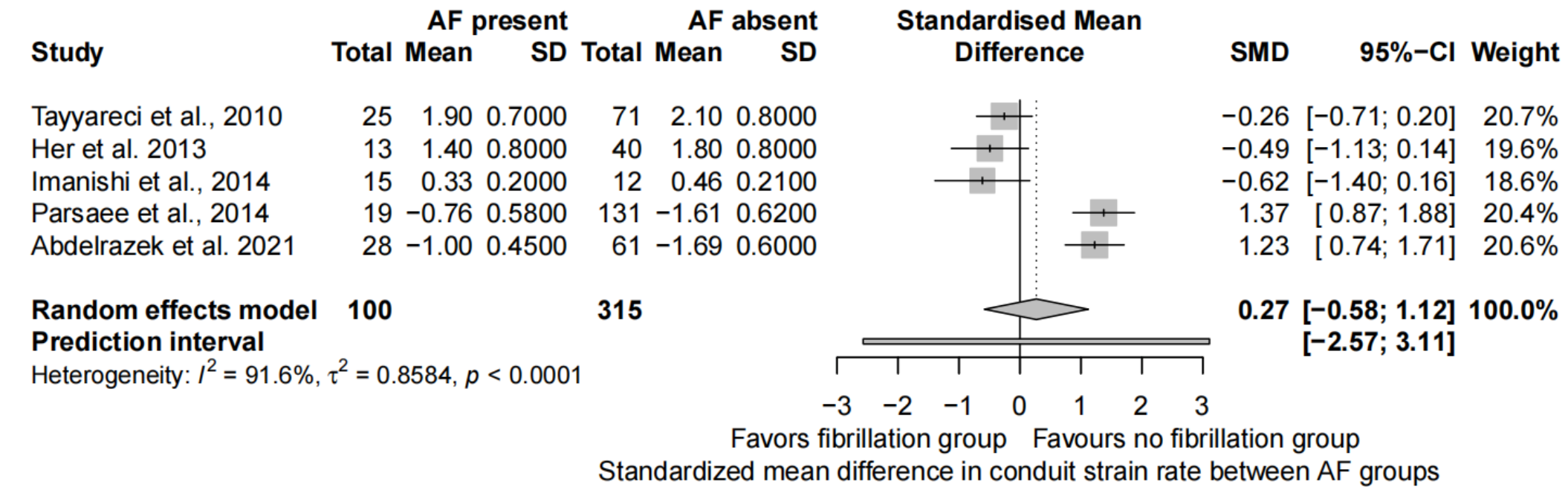

(c)

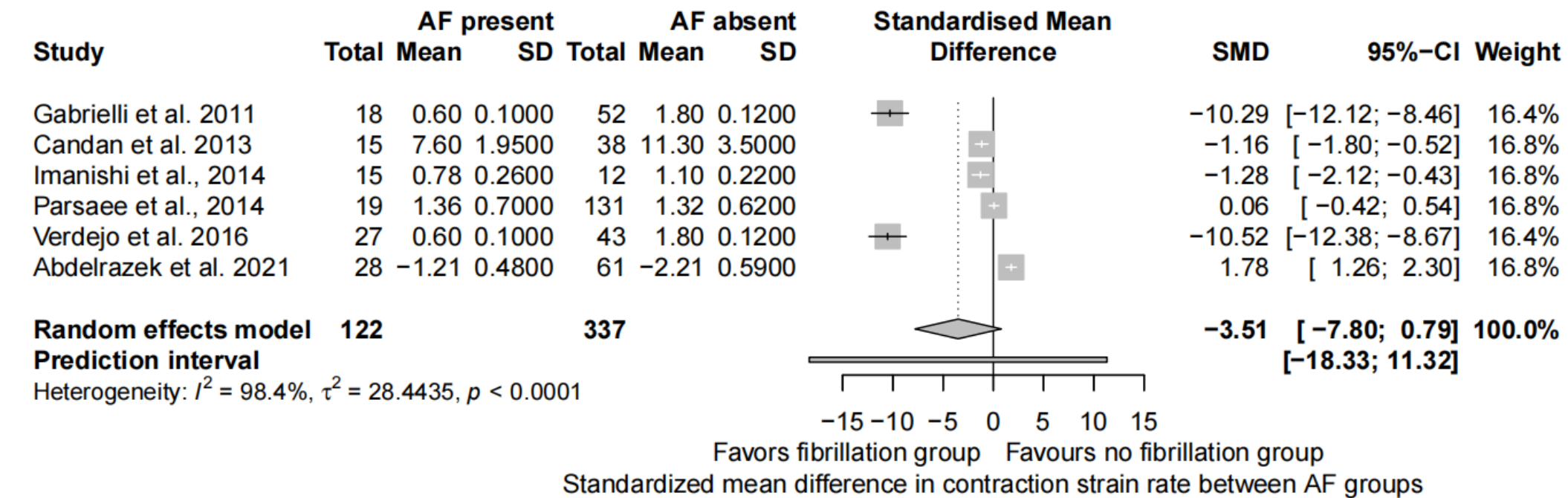

Supplement: ivag035_Supplementary_Data [file ivag035_supplementary_data.zip › Supplementary figure 1.pdf]

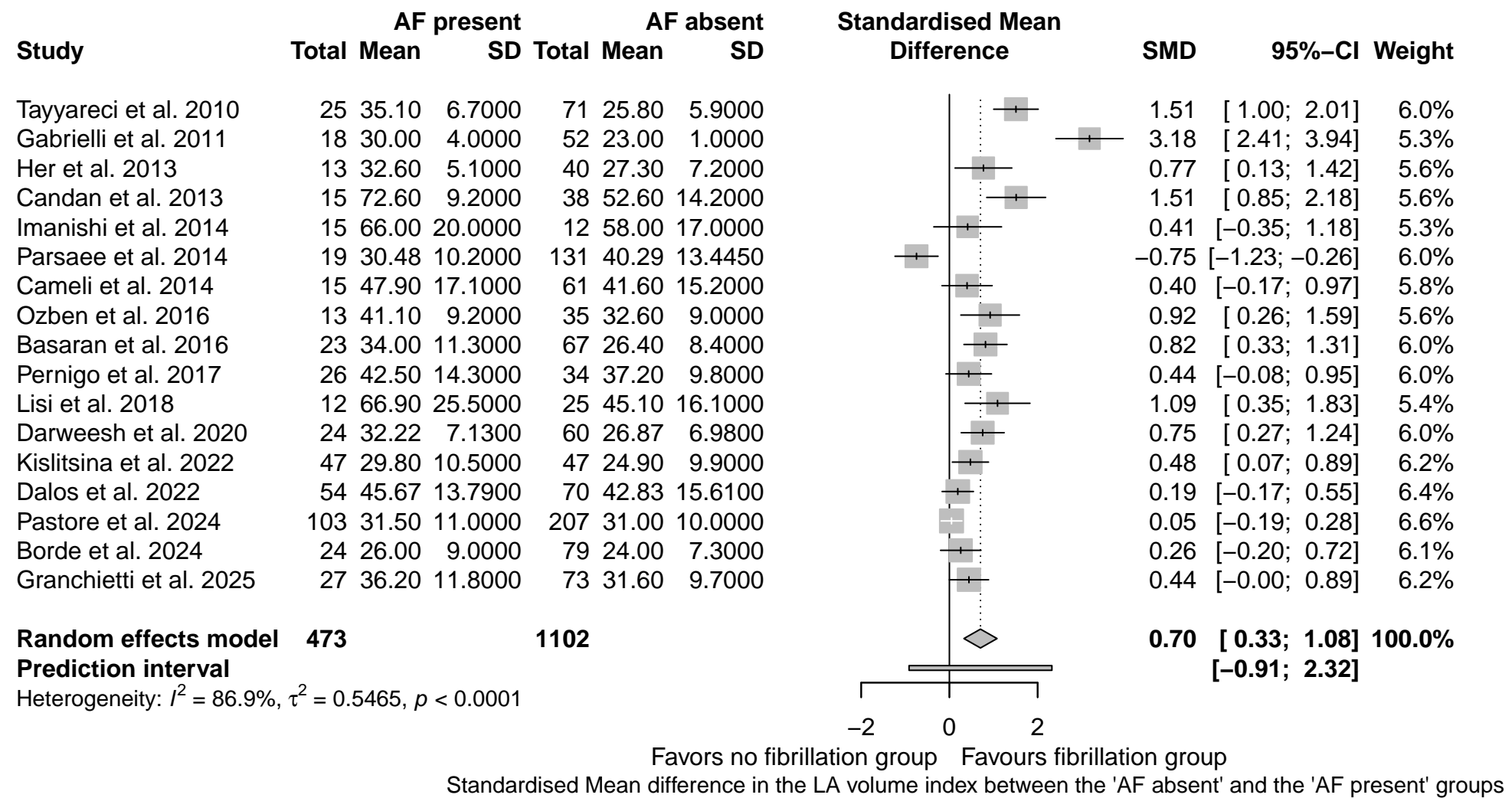

Supplement: ivag035_Supplementary_Data [file ivag035_supplementary_data.zip › Supplementary figure 2.pdf]

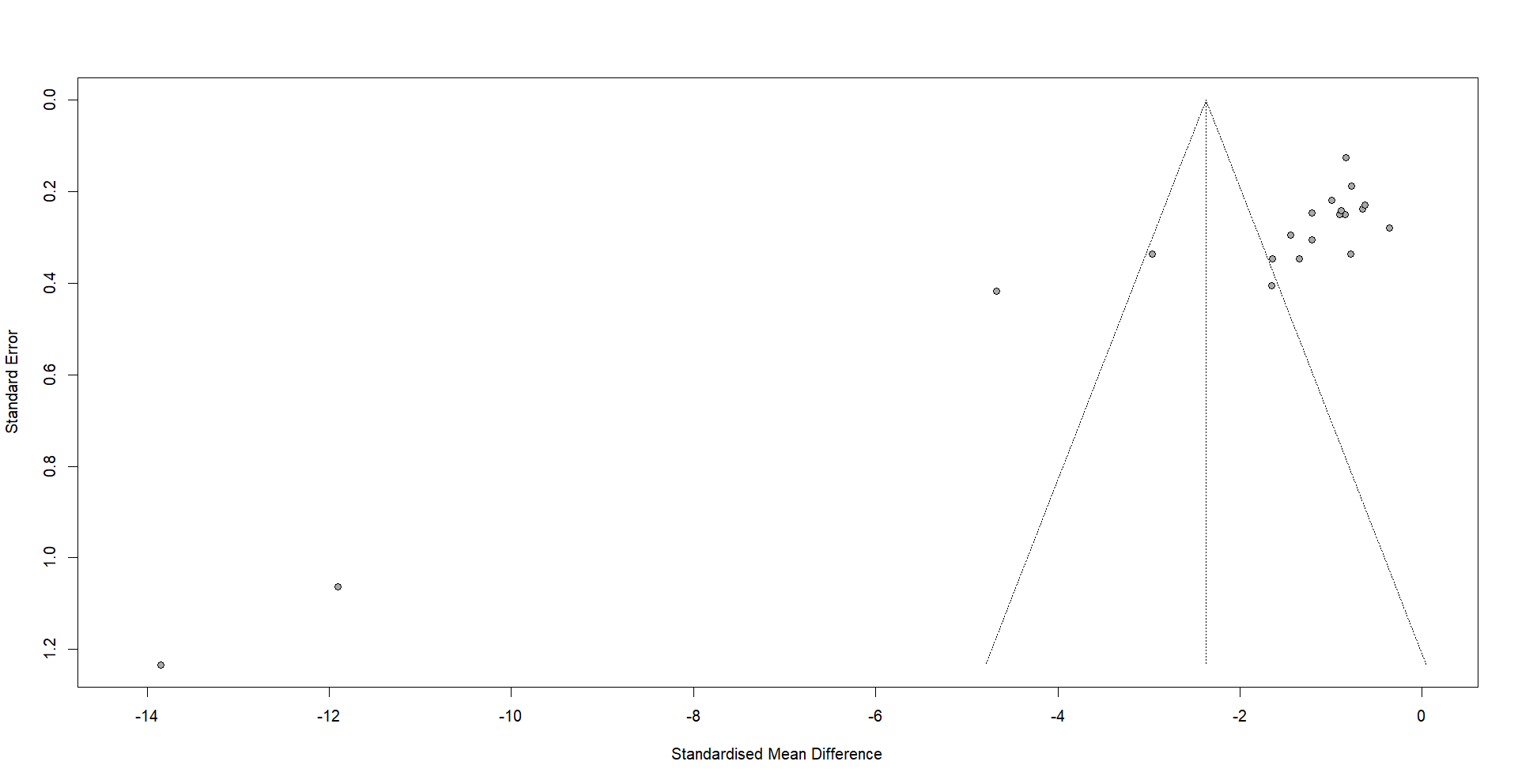

Supplement: ivag035_Supplementary_Data [file ivag035_supplementary_data.zip › Supplementary figure 3.png]
